# The effects of SARS-CoV-2 infection on modulating innate immunity and strategies of combating inflammatory response for COVID-19 therapy

**DOI:** 10.1186/s12929-022-00811-4

**Published:** 2022-05-03

**Authors:** Yiran Wang, Mandi Wu, Yichen Li, Ho Him Yuen, Ming-Liang He

**Affiliations:** 1 Department of Biomedical Sciences, City University of Hong Kong, Hong Kong SAR, China; 2 CityU Shenzhen Research Institute, Nanshan, Shenzhen, China

**Keywords:** SARS-CoV-2, Innate immunity, Inflammatory response, Drug targets

## Abstract

The global pandemic of COVID-19 has caused huge causality and unquantifiable loss of social wealth. The innate immune response is the first line of defense against SARS-CoV-2 infection. However, strong inflammatory response associated with dysregulation of innate immunity causes severe acute respiratory syndrome (SARS) and death. In this review, we update the current knowledge on how SARS-CoV-2 modulates the host innate immune response for its evasion from host defense and its corresponding pathogenesis caused by cytokine storm. We emphasize Type I interferon response and the strategies of evading innate immune defense used by SARS-CoV-2. We also extensively discuss the cells and their function involved in the innate immune response and inflammatory response, as well as the promises and challenges of drugs targeting excessive inflammation for antiviral treatment. This review would help us to figure out the current challenge questions of SARS-CoV-2 infection on innate immunity and directions for future studies.

## Introduction

The entire world is suffering from the COVID-19 outbreak, one of the largest pandemics in human history. This unprecedented crisis, which first emerged in Wuhan, China, in December 2019, was caused by Severe Acute Respiratory Syndrome Coronavirus 2 (SARS-CoV-2). As of April 16, 2022, over 503 million people have been confirmed infections and repeated infections worldwide, leading to approximately 6.22 million deaths. (https://www.worldometers.info/coronavirus/) The patients with SARS-CoV-2 infections exhibit a variety of respiratory symptoms, including fever, fatigue, dry cough, and sore throat, etc. [1]. Severe respiratory symptoms may include Acute Respiratory Distress Syndrome (ARDS) [2]. People with older age and comorbidity, including obesity, diabetes, and cardiovascular disease are more likely to be infected and have severe symptoms [3]. In addition to the elderly, pregnant women and people with other underlying diseases have a higher probability to develop severe disease, due to the physiological immunity change and/or complications.

Besides the current SARS-CoV-2 outbreak, we have experienced three horrible pandemic infections by coronavirus since the twenty-first century. SARS-CoV first broke out in Guangdong province, China, in November 2002. Middle East Respiratory Syndrome Coronavirus (MERS-CoV) occurred in Jordan in 2012. Both coronavirus outbreaks caused extensive severe acute respiratory infectious disease with thousands of death and many more severe complications after recovery [4, 5]. Although patients infected by those coronavirus showed similar symptoms, SARS-CoV-2 displays a much higher transmission rate. R_0_ is the average number of people that each infected person infects if all are susceptible to infectious disease and there is no external interference. The number of new infectious increases exponentially as the outbreak progresses when R_0_ is greater than 1; while large scale transmission could not occur if R_0_ is smaller than 1. Although R_0_ values vary considerably with the stages of the COVID-19 pandemic and in different regions, as compared to the R_0_ value 0.9 of MERS-COV, an average R_0_ value 2.5 for SARS-CoV-2 indicates a dramatic high transmission rate [6, 7]. The variant of SARS-CoV-2, lambda, even has higher infectivity and is resistant to vaccines due to mutation in the N terminal domain (NTD) of spike proteins [8]. People are much easier to be infected by omicron variants because of a lower early doubling time without symptoms in more than 90% of infected individuals as compared to other variants [9]. In this paper, we briefly summarize the findings on the virus structure and life cycle, and update the knowledge on how the virus manipulates the host innate immunity and inflammatory response, the major players of severe acute respiratory disease caused by SARS-CoV-2 infections.

### The structure of SARS-CoV-2

Coronaviruses are enveloped viruses with a positive-sense single-stranded RNA (+ ssRNA) [10]. The structure of coronavirus comprises four major parts: spike protein (S), nucleocapsid protein (N), membrane protein (M), and envelope protein (E). The spike protein is discovered from the outer portion of the virus with 150 kDa molecular weight [11]. The spike protein of SARS-CoV-2 contains a receptor-binding domain (RBD), which recognizes a specific receptor angiotensin-converting enzyme II (ACE2) in host cells [12]. The spike protein is composed of two subunits S1 and S2. The S1 subunit determines its main function in interacting with external proteins. The S1 subunit can be further divided into an N-terminal domain (NTD) and RBD. The interaction between the RBD and the host cell receptor ACE2 is crucial for its entry. The S2 subunit locates in the central region that contributes to the fusion of the virus with cell membrane [11, 13]. The N protein of SARS-CoV-2 binds to and packages the 30 kb RNA viral genome into a ribonucleoprotein complex (RNP) [14]. The modular structure and the dynamic nature of the nucleocapsid protein contribute to its rapid dissociation and exposure of the RNA genome quickly after infection. This explains its efficient transcription and replication under low energy intake, thus playing an important role in SARS-CoV-2 replication in human body [15, 16]. The new variant of SARS-CoV-2, omicron is a recent new threat to the world. The latest studies have shown a large amount of mutation in spike protein, which makes current vaccines and therapy inefficient [17]. Despite the spike and nucleocapsid protein, the M and E proteins also play critical roles in SARS-CoV-2 infection. The M protein, which contains three transmembrane structural domains and one conserved structural domain, is also a component of the viral envelope. It interacts with other membrane proteins to stabilize nucleocapsid protein in the process of virion assembly [18]. Despite being the smallest structural protein in coronavirus, the envelope protein contains a hydrophobic structural domain and a transmembrane α-helix domain. The E protein also plays an important role in the life cycle, including budding, assembly, and envelope formation [19]. Besides the unique role of each protein, they work together to make SARS-CoV-2 like a sophisticated instrument.

### SARS-CoV-2 reproduction and transmission

The process of SARS-CoV-2 infection is similar to SARS-CoV and MERS-CoV. ACE2 works as an essential receptor through which SARS-CoV-2 enters the host cell by direct binding to the spike protein [20]. ACE2 has been reported to be expressed in the lungs, nasal, oral mucosa, and gastrointestinal tract. Studies have shown that the RBD of SARS-CoV-2 has a higher affinity to ACE2 than that of SARS-CoV [21]. A structural analysis reveals different biochemical components at the binding site between SARS-CoV-2 and SARS-CoV RBD regions, leading to a higher affinity of SARS-CoV-2 spike on ACE2. On the other hand, the residue change caused by single nucleotide polymorphisms (SNP) of ACE2 may increase the affinity of ACE2 to spike protein as shown by a silico molecular docking simulation [22]. The higher affinity may be one of the reasons why SARS-CoV-2 can spread more easily and has a higher transmission ability. A recent study has shown that SARS-CoV-2 also infects ACE2-deficient T cells. Ectopic expression of CD147 promotes virus entry into T cells and other ACE2-deficient cells [23], suggesting a potential new drug target for COVID-19 therapeutics.

**Fig. 1:**
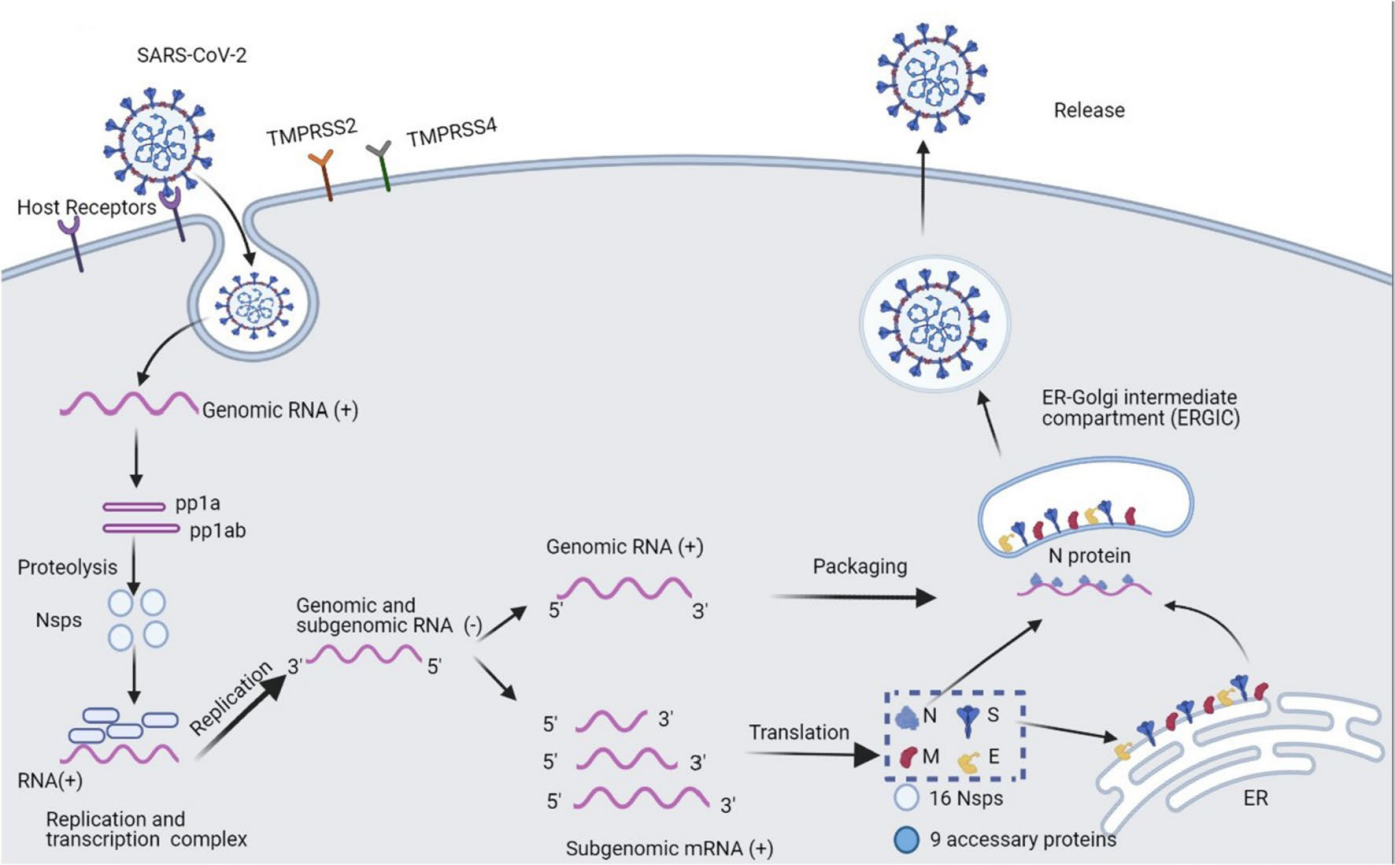
The entry and RNA replication of SARS-CoV-2 [30]. SARS-CoV-2 enters the host cell by interaction between its spike protein and ACE receptor. Next, the viral RNA is released and translated into polyproteins, which is then cleaved into Nsps. RNA replicates from RTC to the negative-stranded RNA (replication intermediate), which can further be transcribed into positive-stranded RNA genomic RNA (also functioning as mRNA) and subgenomic mRNAs from the replication intermediates and further translated into structure proteins. The new virion is packaged in ERGIC and finally released outside of the host cells. TMPRSS, transmembrane serine protease; Nsp, nonstructural protein; RdRp, RNA-dependent RNA polymerase; ERGIC, ER-Golgi intermediate compartment. (Created with BioRender.com.)

Once the spike protein is bound to ACE2, the host proteases are involved in the cleavage of the spike protein to activate the entry of SARS-CoV-2. Type II and type IV transmembrane serine protease (TMPRSS2/TMPRSS4) assist the virus to enter the cell by facilitating spike fusogenic activity and by cleaving the spike protein. The spike protein helps to fuse the envelope of SARS-CoV-2 with the cell membrane of alveolar type 2 cells through S1 binding to the glucose regulated protein 78 (GRP78) receptor, resulting in structural changes in the S2 subunit and RBD opening. These processes allow better binding of spike proteins to the receptors on the cell membrane [24]. When the virus enters host cells, RNA is released into the cytoplasm and translated into two polyproteins: pp1a and pp1ab. These two proteins are divided into 16 non-structural proteins (Nsps) that recruit membrane structures from the host cell, aggregate replication and transcription complex (RTC), and then generate antisense negative-stranded subgenomic RNAs. There are two further processes: 1) the sense strand RNA is produced by replication, and repackaged in the later viruses, and 2) different lengths of subgenomic mRNA are synthesized by discontinuous transcription through RNA-dependent RNA polymerase (RdRp) binding and initiating transcription at different points on the antisense template [25]. After translation, structural proteins (S, M, E, and N) and viral genome are assembled into virions in the ER-Golgi intermediate compartment (ERGIC). This helps the new virion to fuse with the plasma membrane and be released from the host cells (Fig. 1), seeking other cells as potential targets of infection [26]. For example, SARS-CoV-2 is normally reproduced in the respiratory tract, while the transmission occurs through the saliva and other body fluids of infected individuals. Coughing, sneezing, and even breathing can lead to the spread of saliva droplets and the infection of SARS-CoV-2 [27]. The transmission is influenced by distance and usually smaller droplets travel longer distances because gravity does not affect them too much [28]. The virus can then enter the mouth, eyes, and nose of a susceptible individual and start to reproduce. The risk of infection can be lowered by wearing a facial mask, eye shield, and maintaining a social distance [29].

### Innate immunity and inflammatory response

#### Recognition

Innate immunity is the front defense line for combating pathogen invasion and critical for activation of adaptive immunity. To sense pathogen invasion, mammalian cells have evolved several pattern-recognition receptors (PRRs), including Toll-like receptors (TLRs), C-type lectin receptors (CLRs), NOD-like receptors (NLRs), RIG-I-like receptors (RLRs), and the AIM2-like receptors (ALRs) [31]. These receptors recognize either pathogen-associated molecular patterns (PAMPs) or cellular damage caused by pathogens (damage-associated molecular patterns DAMPs). When PRRs are activated, leukocyte recruitment and a strong inflammatory response will be generated and promoted [32].

**Fig. 2:**
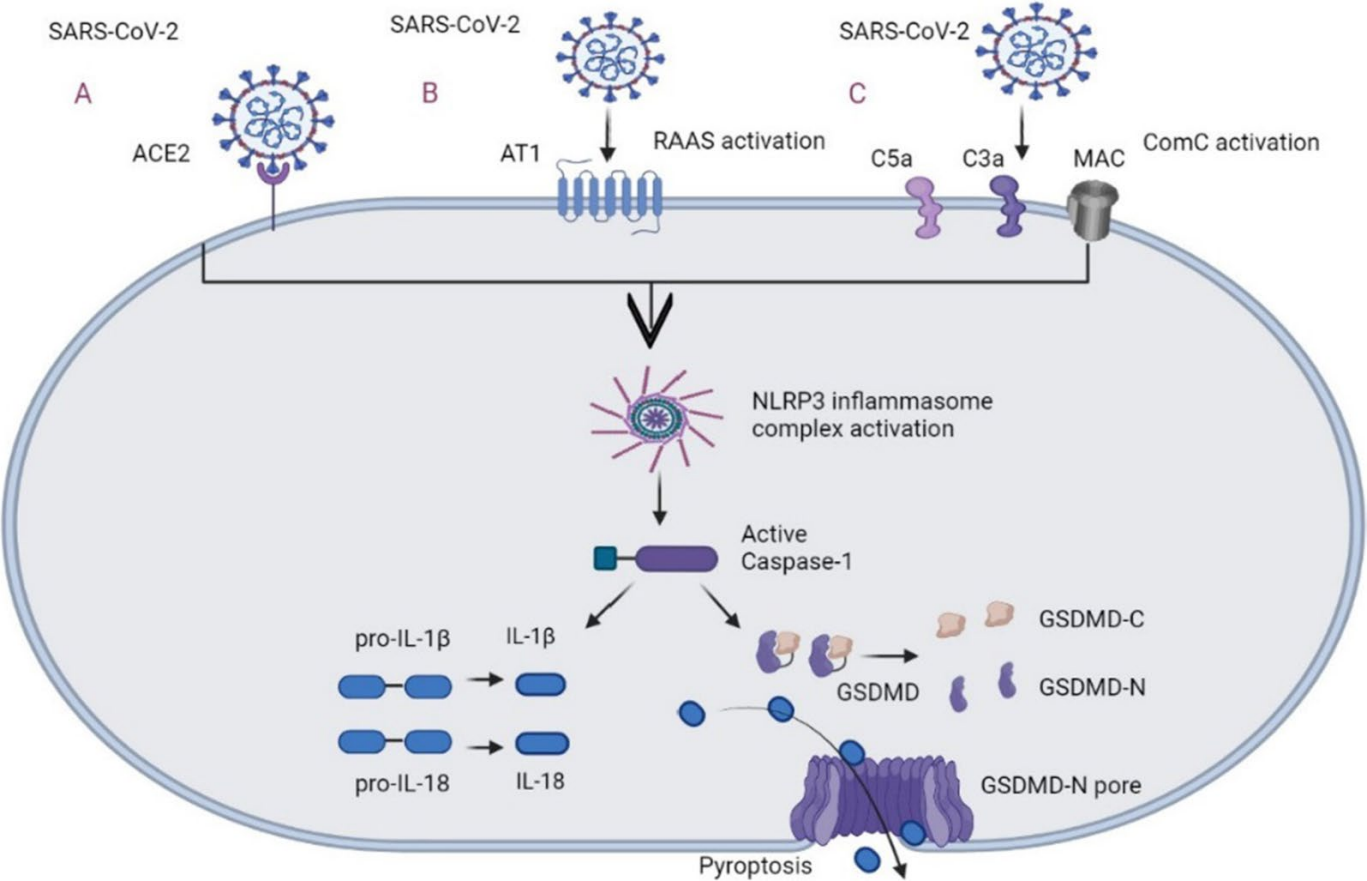
The activation and effect of NLRP3 [36, 37]. The activation of NLRP3 can be realized through directly binding to the receptor, or through RAAS, and ComC. Subsequently, NLRP3 assemble and active caspase-1 further allow IL-1β and IL-18 to release. ACE2, angiotensin converting enzyme; RAAS, renin–angiotensin–aldosterone system; ComC, complement cascade; GSDMD, gasdermin D. (Created with BioRender.com.)

In response to DAMPs and PAMPs, inflammasomes can be assembled by some PRRs. The inflammasome is a macromolecular complex that consists of receptors, caspase, inclusive or exclusive caspase activation and recruitment domain (ASC) [33]. The inflammasome is known for its ability to defend against pathogens by punching holes in membranes and releasing cytokines that lead to cell death in the form of inflammation known as pyroptosis [34]. The inflammasome may serve as a biomarker to indicate the severity of diseases. Inflammatory molecules could serve as ideal targets for the treatment of patients infected with SARS-CoV-2 [35]. NLR family pyrin domain containing 3 (NLRP3), a well-studied inflammasome, can cleave and activate essential inflammatory molecules, such as cleavage of pro-caspase-1 to release active caspase-1. A study has identified three possible pathways for NLRP3 activation in response to SARS-CoV-2 (Fig. 2). NLRP3 inflammasome is directly activated after spike protein binds to ACE2 in the host cells. Alternatively, the level of angiotensin II is increased due to the activation of the renin–angiotensin–aldosterone system (RAAS). The NLRP3 inflammasome levels increase after angiotensin binding to the A1 receptor. The last pathway is the recognition and activation of the complement cascade (ComC) through the Mannan-binding lectin (MBL)/MBL-associated serine protease-2 (MASP-2) complex, leading to the release of ComC cleavage fragment (C3a and C5a) and the C5b/C9 membrane attack complex (MAC) that activate NLRP3 [36]. Upon oligomerization and activation of NLRP3, caspase-1 is incorporated and becomes an active form. The activated caspase-1 cleaves pro-IL-1β and pro-IL-18 into IL-1β and IL-18, and gasdermin D (GSDMD) into N and C terminus. The N terminus (GSDMD-N) then forms pores in the cell membrane, thereby allowing the release of IL-1β and IL-18. This process promotes pyroptosis, especially in lymphocytes and macrophages [37, 38]. A recent study has shown that the nucleocapsid protein of SARS-CoV-2 can bind and help NLRP3 assembly by enhancing the interaction between NLPR3 and ASC [39]. The level of the NLRP3 inflammasome may explain the relationship between comorbidity and SARS-CoV-2 infection. For example, people with obesity already have pre-existing inflammatory state through NLRP3. With the infection of SARS-CoV-2, the pro-inflammatory response may be promoted and other degenerative chronic comorbidities can be developed, leading to a higher risk [38].

#### Cytokine storm

Large amounts of cytokines are commonly observed in the blood of many COVID-19 patients [40]. Cytokines can be released by infected somatic cells, inflammatory macrophages, and other kind of lymphocytes. In macrophage and epithelial cells, the activation of inflammasome (such as NLRP3) releases pro-inflammatory cytokines, including IL-1β and IL-18. After sensed viral RNA by Toll-like receptors (TLR3, TLR7, TLR8, and TLR9). The signaling of these cytokines activates nuclear factor-κB (NF-κB) to further promote pro-inflammatory cytokine expression and release [34, 41]. Normally, the important function of released cytokine is to facilitate tissue repair and defend the pathogen invasion. However, a large amount of cytokine release at the same time is harmful. The aberrant situation is known as cytokine storm that damages tissues by destroying the protective lining of blood vessels, a possible main reason for ARDS in patients [42]. Other viruses, such as SARS-CoV, MERS-CoV, influenza virus, enterovirus A71, and Ebola, were also reported to induce cytokine storms and cause mortality [43–46]. Studies on SARS and MERS-CoV patients have demonstrated strong correlations among the increased pro-inflammatory factors, tissue damage and severe inflammation [47]. A great level change of cytokines and chemokines were observed in severe cases, including, IL-6, IL-1β, IL-2, IL-7, IL-12, CXCL8, CXCL9, and CXCL10 [48–50]. Moreover, SARS-CoV-2 induces a large number of cytokines at an earlier stage as compared with SAR-CoV and MERS, probably explaining why symptoms of patients with severe infection aggravate in a short time [51].

**Fig. 3:**
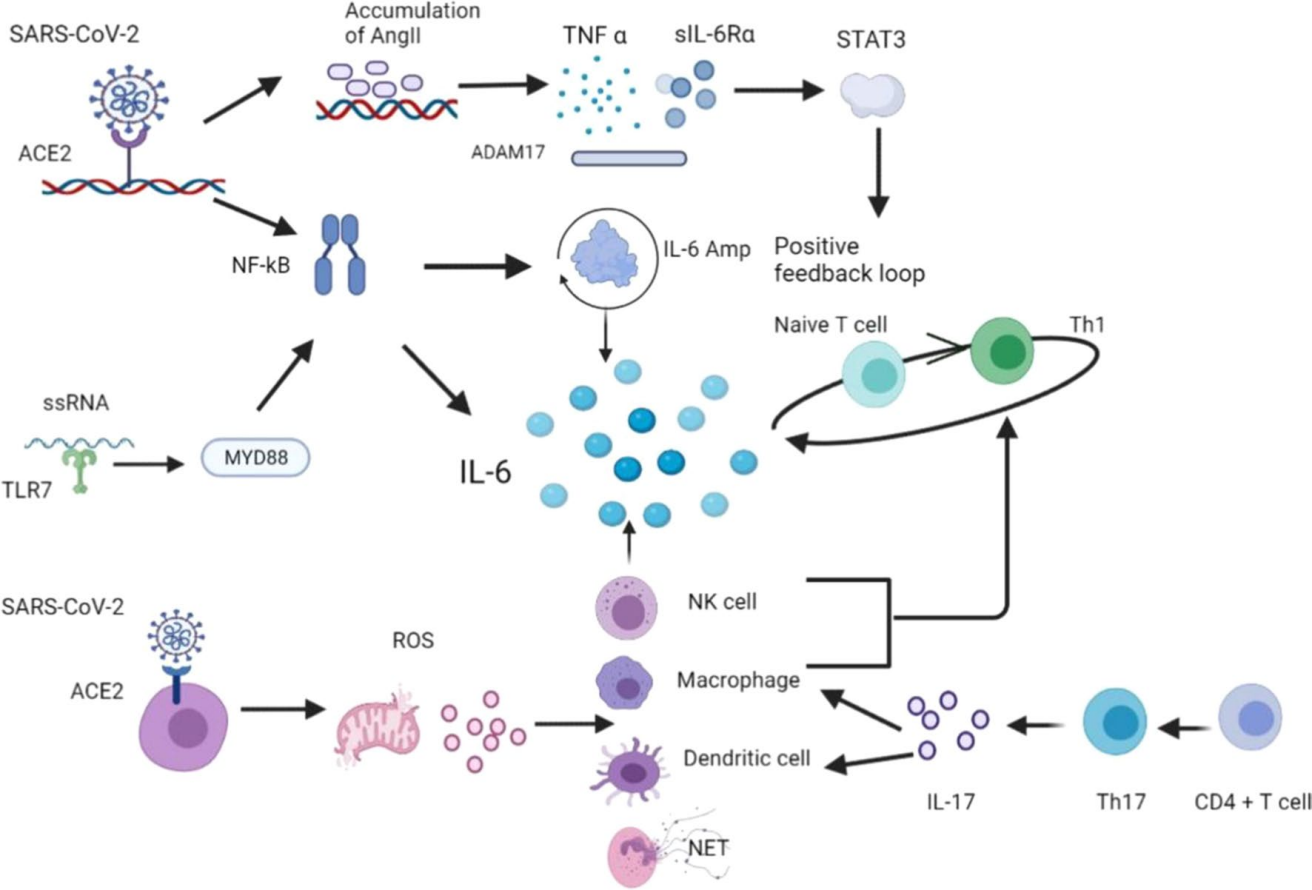
Signaling pathways involved in the production and release of IL-6 upon SARS-CoV-2 infections [56, 57]. IL-6 can be released after activation of different pathways, including TLR7, Ang II and STAT3 signaling pathways. Several types of cells, such as NK cells and macrophages, produce more IL-6 through activation of ROS. TLR7, toll-like receptor 7; Amp, IL-6 amplifier; ADAM17, disintegrin and metalloprotease 17; Th17, helper T17; MYD88, myeloid differentiation primary response protein 88; STAT3, signal transducer and activator of transcription 3; Ang II, angiotensin II; NF-κB, nuclear factor-κB; (Created with BioRender.com.)

IL-6 is considered as an important indicator for cytokine storms. A high level of IL-6 are observed in non-surviving patients and it is also associated with ARDS [52, 53]. IL-6 can be produced and released by several different mechanisms, most commonly through TLR- and RLR-signaling cascades (Fig. 3). TLR7 recognizes viral ssRNA and then recruits myeloid differentiation primary response protein 88 (MYD88), triggering transcription factors (TFs, such as interferon regulatory factor 7 (IRF7) and NF-κB) translocation into nucleus to transcribe mRNAs of many cytokines, including IL-6 [54, 55]. Another potential way is through the angiotensin II associated pathway. SARS-CoV-2 activates NF-κB via PRRs. When the virus binds to ACE2, the expression of angiotensin II (Ang II) is increased due to the occupancy of ACE2. The accumulation of Ang II also induces cytokines, such as tumor necrosis factor alpha (TNFα) and soluble IL-6 receptor forms (sIL-6Rα) via disintegrin and metalloprotease 17 (ADAM17), which subsequently activate signal transducer and activator of transcription 3 (STAT3). Additionally, cells that mediate adaptive immunity are also involved in this process. The IL-6 amplifier releases several pro-inflammatory cytokines, including IL-6. They form a positive feedback loop by recruiting other cells, including macrophage and activated T cells [56]. IL-6 is also produced by the infiltration of macrophages, dendritic cells, NK cells and neutrophils in response to reactive oxygen species (ROS) [57]. IL-6 further skews naïve T cells toward T helper type I (Th1) cells, which release more cytokines including IL-6, and create a positive feedback loop [58]. Moreover, the interaction between natural killer cells and dendritic cells can initiate T cell immunity and make naïve T cells prone to Th1 [59]. Besides, the activated CD4^+^ T cell may differentiate into Th17 cells with the help of IL-6 and TGFβ. Th17 cells subsequently releases IL-17, which then targets macrophages and dendritic cells for further IL-6 production [59].

#### Interferon release

**Fig. 4:**
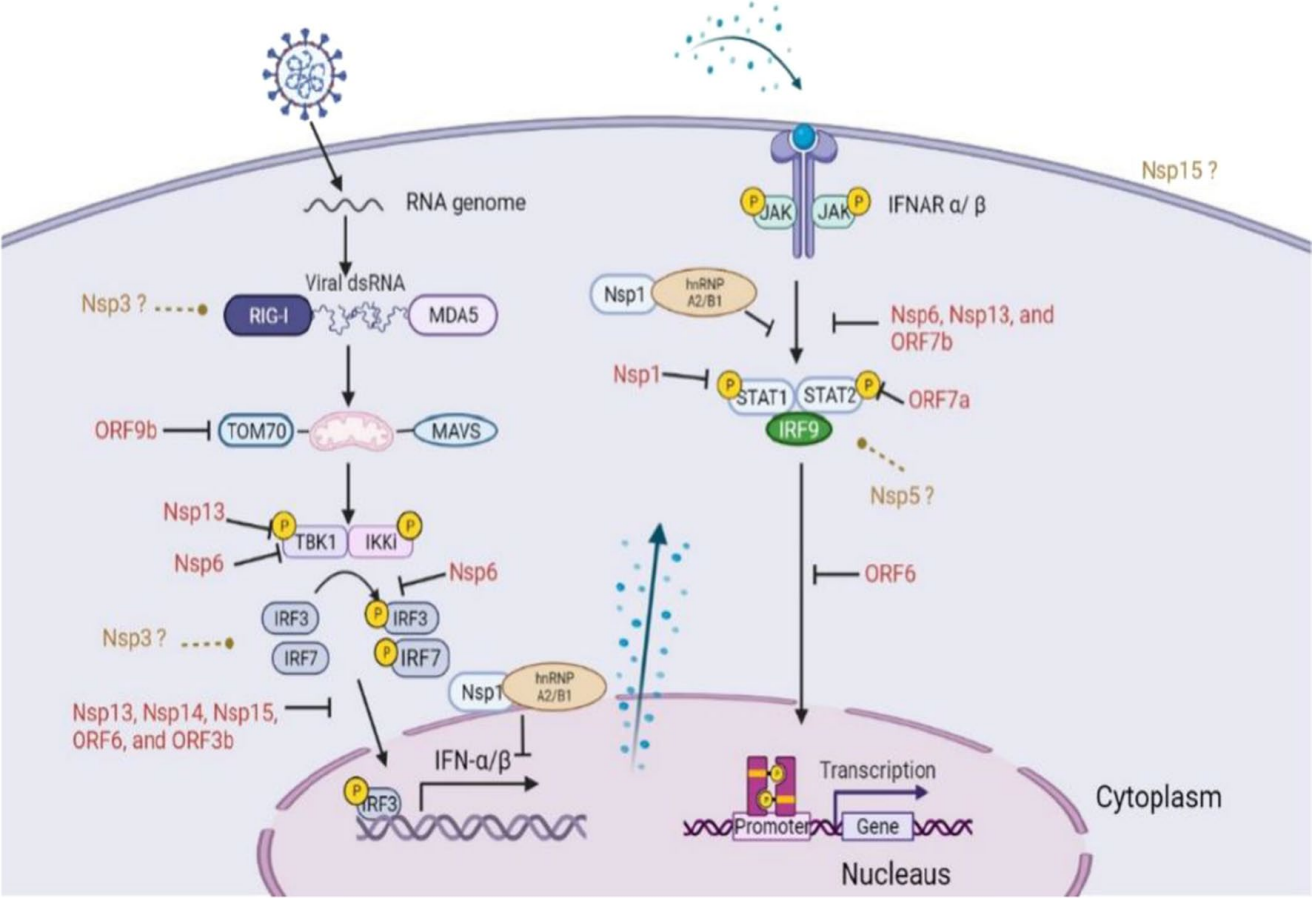
Antagonism of type I interferon by SARS CoV-2 proteins. Interferon responses are triggered by SARS-CoV-2 RNA. The nonstructural protein of SARS-CoV-2 target different proteins and inhibit their anti-inflammatory interferon response. Potential proteins and targets needed to be further studied are shown in brown question marks. MDA5, melanoma differentiation-associated protein 5; TOM70, translocases of outer membrane 70; TBK1, TANK binding kinase 1; IKKɛ, inhibitor of k-B kinase-ɛ; IFN, interferon; IRF, interferon regulatory factor; IFNAR, interferon-α/β receptor; STAT, signal transducer and activator of transcription; Nsp, nonstructural protein; ORF, open reading frames. (Created with BioRender.com.)

Among cytokines, type I interferons (IFN-α/β) play critical roles in innate immunity and protect host cells from viral infections. They exhibit significant effects on the control of pathogen spread by inducing cell-intrinsic antimicrobial states, managing and balancing the innate immunity responses, and activating adaptive immunity including T cells and B cells activities [60]. It was found that RLRs, retinoic acid-inducible gene I (RIG-I) and melanoma differentiation-associated protein 5 (MDA5), are activated upon recognizing the genome RNA of SRAS-CoV-2 during viral replication. The activated RLRs bind to mitochondrial antiviral signaling protein (MAVS) via caspase activation and recruitment domain (CARD)-CARD interaction [61]. MAVS is then activated to recruit TANK binding kinase 1 (TBK1) and inhibitor of NF-κB kinase-ɛ (IKKɛ), which are responsible for the phosphorylation of interferon regulatory factor 3 and 7 (IRF3 and IRF7) [62]. Subsequently, the phosphorylated IRF3 and IRF7 dimerize and translocate to the nucleus. This process promotes the transcription of IFNs and IFN-stimulated genes (ISGs). IFNs further induces more IFNs and ISGs expression via the Janus kinase (JAK)-STAT pathway by allowing STAT to translocate to the nucleus (Fig. 4) [63]. Moreover, NF-κB can be activated via IKK and translocated to the nucleus after the activation of the RLR receptors. This promotes the expression of genes that code for pro-inflammatory proteins [64]. A decreased expression level of methyltransferase-like 3 (METTL3) is discovered in patients with severe SARS-CoV-2 infection. The downregulated METTL3 decreases m6A modification in the 3’ end genome of the virus, which in turn increases RIG-I binding and stimulates innate immune response [65].

#### Strategies of SARS-CoV2 to evade innate immunity

Although the host has developed very complicated and effective innate immunity strategies to combat pathogen invasion, SARS-CoV-2 evolves their own ways to attenuate the innate immunity for escape of host defense. Among those strategies, type I interferon response is one of the most effective ways to limit and even eradicate the invaded pathogens. In the following section, we will discuss the evasion strategies for antagonizing type I interferon signaling as well as other strategies used by SARS-CoV-2.

#### SARS-CoV-2 antagonizes IFN-α/β induction and signaling

In severe patients, researchers have found type I interferon deficiency and impaired response in their bodies [66]. The impaired innate immunity may be probably caused by viral proteins by antagonizing type I IFN induction and the downstream signaling [67]. Nonstructural proteins of SARS-CoV-2 play important roles to disturb IFN-α/β by several mechanisms through the interferon signaling pathway, resulting in successful viral replication and transmission [68]. Sixteen Nsps encoded by SARS-CoV-2 form RTC and help the virus to replicate [69]. In this review, we will first discuss some nonstructural proteins and their functions in repressing IFN-α/β response.

Nsps block the induction of type I interferon production. Nsp13 belongs to SF1 helicase superfamily and is a component of RTC [70]. Nsp13 of SARS-CoV2 shares a very high identity with SARS-CoV, which highlights its importance for viral replication [71]. It has been revealed that Nsp13 acts as an IFN antagonist [72]. Xia and colleagues have shown that Nsp13 can bind to TBK1 and prevent its phosphorylation, which further disturbs IFN-α/β response [73]. Nsp6 is also demonstrated to bind with TBK1. However, Nsp6 does not affect TBK1 phosphorylation, showing a different mechanism from Nsp13. The interaction between Nsp6 and TBK1 decreases the phosphorylation of IRF3 and nuclear translocation, leading to a decreased IFN-α/β production. Other Nsps, including Nsp13, Nsp14, Nsp5 and Nsp15, are also found to block IRF3 nuclear translocation, and further prevent IFN-α/β expression [74, 75]. Nsp14, which mediates guanine N7-Methylation of 5′ cap, is another strategy for virus evasion of host innate immunity during SARS-CoV-2 infection. Nsp14 mutation decreases N7-methyltransferase activity in some SARS-CoV2 mutant strains, which are shown less replication ability in mice models [76].

Nsps also repress type I IFN signaling through STAT signaling. Nsp1 can promote host mRNA degradation and suppress host protein translation [77, 78]. SARS-CoV-2 Nsp1 inhibits the phosphorylation of STAT1, while Nsp6 and Nsp13 repress both STAT1 and STAT2 phosphorylation [73]. Recently, it has been demonstrated that NSP1 binds to the heterogeneous nuclear ribonucleoprotein (hnRNP) A2/B1 and inhibits the translocation of hnRNP from cytosol to nucleus. The binding also decreases the phosphorylation level of STAT1 and STAT2 that impedes innate immunity [79]. Another study has shown that Nsp5, the main protease of SARS CoV-2, inhibits JAK-STAT signaling and prevents the production of IFN and ISGs by promoting STAT1 degradation [80].

Except nonstructural proteins, accessory and structural proteins also play important roles in inhibiting type I interferon response. Studies have shown that ORF6, ORF8, ORF3b, ORF7a, ORF7b, and nucleocapsid protein potently inhibit the production of interferons as well as their signaling [81]. Specifically, ORF6 inhibits the translocation of IRF3 and STAT signaling [73, 74]. ORF7a blocks the STAT2 phosphorylation, while ORF7b inhibits both the STAT1 and STAT2 phosphorylation [73]. Additionally, ORF9b represses IFN-α/β expression by association with translocases of outer membrane 70 (TOM70), an important adaptor linking MAVS to TBK1/IRF3 [82–84]. ORF9b inhibits the IRF3 phosphorylation and translocation through interacting with RIG-I, MDA5, and TBK1 [85]. An upregulated ORF9b is discovered in Alpha variant, suggesting a better evasion strategy and higher transmission [86]. ORF3b is also considered to restrain IRF3 nuclear translocation (Fig. 4). ORF3b of SARS-CoV-2 represses IFN-α/β expression more potently than that of SARS-CoV [87]. In addition, M protein can also target RIG-I and MDA5 signaling to impede type I interferon induction [88]. N protein is also considered as a potent inhibitory factor of innate immune response. The dimerization domain of N protein is required for liquid–liquid phase separation (LLPS), and inhibits type I interferon production by influencing MAVS aggregation [89].

#### SARS-CoV-2 antagonizes other pathways

Except for inhibiting IFN-α/β response, SARA-CoV-2 evolves other strategies to facilitate its replication, spread and infection. Some of the SARS-CoV-2 proteins target and inhibit NF-κB pathway. Nsp13 can moderate NF-κB phosphorylation and nuclear translocation [90]. It has been reported Nsp3 of SARS-CoV stabilizes IκBα, an inhibitor of NF-κB, to prevent NF-κB signaling pathway [91]. More research is still needed on SARS-CoV-2. In addition, Nsp1 and Nsp13 are regarded as NLRP3 inflammasome antagonists. They repress NLRP3 inflammasome induced caspase-1 activity and the secretion of IL-1β [92]. On the other hand, alveolar macrophages cannot sense SARS-CoV-2 and do not produce interferon response or ISGs after challenging with SARS-CoV-2. This could be an explanation of asymptotic phase at initial infection stages [93]. Interestingly, the interaction between ORF8 and major histocompatibility complex (MHC I) suppresses the innate immunity by triggering lysosome-dependent MHC-1 degradation [94].

#### Cells participate in innate immune and inflammatory response

An analysis of bronchoalveolar lavage fluids (BALF) of COVID-19 patients showed a different cluster of cells, such as neutrophils, NK cells, macrophages, and epithelial cells. As compared to healthy people, high levels of macrophages and neutrophils are also discovered in BALF of severe patients infected by SARS-CoV-2 [95]. These cells also play important roles in eliciting an inflammatory response. In the following paragraphs, we plan to explain their different roles in SARS-CoV-2 infection.

#### Alveolar epithelial cells and pulmonary vasculature

Alveolar epithelial cells are categorized as type I and II epithelial cells. Type I alveolar epithelial cells cover about 90% of epithelial cells in lung. Although only a small proportion belongs to type II alveolar epithelial cells, they play important roles in anti-inflammatory response and preventing ARDS [96]. High level of ACE2 and TMPRSS2 expression is found mainly in type II alveolar epithelial cells in COVID-19 patients, indicating the epithelial cells are more susceptible for infection [97, 98]. During the viral replication process, the double-stranded RNAs (dsRNAs) of SARS-CoV-2 can initiate antiviral effects. When PRRs sense viral dsRNAs, three signaling pathways, including IFN signaling, oligoadenylate synthetase–ribonuclease L (OAS-RNase L), and protein kinase R (PKR) are activated. RIG-I, MDA5, and laboratory of genetics and physiology 2 (LGP2) are commonly involved in the induction of IFN production. All three proteins contain a carboxy-terminal domain (CTD) and a helicase domain, while RIG-I and MDA5 have two extra amino-terminal CARD domains [99]. A recent study has shown that MDA5 and LGP2, but not RIG-I, induce IFN response in lung epithelial cells. Except for RLR, NOD1 is also required for recognizing viral RNA in lung epithelial cells to initiate IFN response. Moreover, IRF3 and IRF5, but not IRF7, are important in IFN induction in SARS-CoV-2 infected lung epithelial cells [100]. Nevertheless, another study revealed a low level of IFN response in infected lung epithelial cells. This is probably caused by viral proteins mediated inhibition of IFN response. However, OAS-RNase L and PKR levels are obvious in lung epithelial cells [101]. Through analyzing differentially expressed genes (DEBs) of lung epithelial cells infected by SARS-CoV, SARS-CoV-2, and MERS-CoV, SARS-CoV-2 suppressed type I interferon production in a much stronger way. Quite a few genes involved in type I interferon signaling, such as IRF1, STAT1, and ISGs, are downregulated much more in lung epithelial cells infected by SARS-CoV-2, as compared to the infections by other two coronaviruses [102].

Results from recent studies show that the activation and dysfunction of pulmonary endothelium are hallmarks and the main pathological causes of ARDS in COVID-19 patients. The SARS-CoV-2 infection causes damages to pulmonary vasculature. Although the detailed underlying mechanism is to be extensively studied, it may be medicated by direct tropism, promoting hypercoagulative state, triggering inflammation, and even forming new blood vessels [103]. Obviously, it would be an important research direction to elucidate how SARS-CoV-2 infection damages the physiological structure of the pulmonary vasculature and its microenvironment for induction of ARDS.

#### Neutrophils

**Fig. 5:**
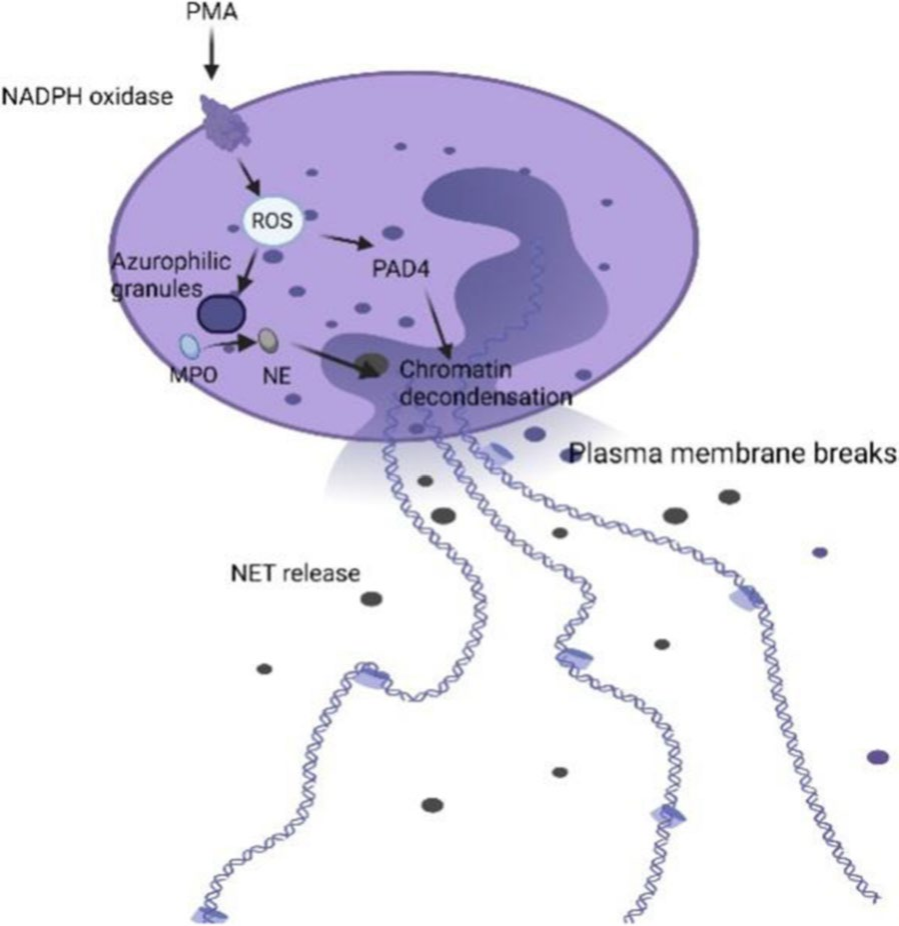
The mechanism of neutrophil extracellular traps (NETs) [105]. NET is triggered by ROS. NE is translocated from azurophilic granules to the nucleus by MPO. NET releases after breakage of the plasma membrane. ROS, reactive oxygen species; NE, neutrophil elastase; MPO, myeloperoxidase; PAD4, peptidyl arginine deiminase 4; NETs, neutrophil extracellular traps. (Created with BioRender.com.)

Neutrophils account for 50–70% of leukocytes. They are critical responding cells in the innate immune system. High levels of neutrophils in the body can cause diseases with obvious symptoms, such as fever, chills, and sweating. Therefore, it may serve as a predictive death marker in severe COVID-19 patients. The SARS-CoV-2 infection could significantly activate neutrophils levels [104]. Neutrophils kill pathogens in three main ways: phagocytosis, degranulation, and form neutrophil extracellular traps (NETs) [105, 106]. NETs are a complex of histones, DNA, and granule proteins released by neutrophils. NETs can effectively neutralize invasive microorganisms through the induction of a specific type of cell death (NETosis) [107]. Higher levels of NETs are found by testing the levels of myeloperoxidase (MPO)/DNA complexes in the plasma of COVID-19 patients [108, 109]. In the process of NETosis, the nuclei of neutrophils lose shape and the cell membrane disassemble. The mechanism of NETs is tightly related to the generation of ROS by NADPH oxidase. Histones are citrullinated by peptidyl arginine deiminase 4 (PAD4), while MPO activates and translocates neutrophil elastase (NE) from azurophilic granules to the nucleus, contributing to chromatin decondensation. Subsequently, the plasma membrane ruptures, allowing NET release in the extracellular space (Fig. 5) [105, 110, 111]. Research has indicated that the upregulated level of IL-1β, IL-6, TNFα, CXCL8, CCL20, CCL2, CCL3, and CCL4 are related to NET production and neutrophils activation [112, 113].

Although NETs are important barriers to protect human bodies, uncontrolled NETs are harmful and probably related to ARDS [109]. The inability of NETs to perform efferocytosis, a process by which phagocytes clear apoptotic cells, could cause persistent inflammation in ARDS. Restoration of AMPK (AMP-activated protein kinase) activity increases efferocytosis and helps to decrease inflammatory lung injury [114]. Metformin, as an activator of AMPK, may increase efferocytosis and serve as a potential strategy to reduce the severity of ARDS [115]. Moreover, hyperactivated state of neutrophils marked by upregulated IL-1β, CXCL8, and S100A12 were found in SARS-CoV-2 infected patients [113]. However, the expression of CD274 only existed in severe cases and the proportion increased in the late stage. The researchers indicated that neutrophils probably be suppressive in severe COVID-19 patients, because CD274^+^ neutrophils can suppress T cell functions [116]. Peripheral blood mononuclear cells (PBMC) in severely infected patients also contain immature neutrophils compared to mild cases [116]. However, another study showed an increased low-density neutrophils level in children and adults who tested negative but had exposed to SARS-CoV-2, suggesting that this probably serves as a protective strategy. An important finding is that the infected children had an increased level of CD63^+^ neutrophils when they were in the acute phase [117].

#### Macrophage

**Fig. 6:**
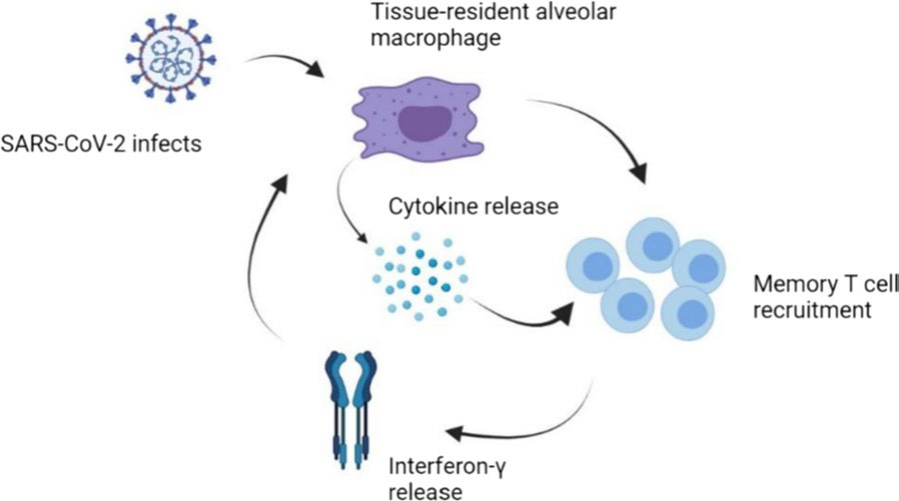
A positive feedback loop between infected macrophages and T cells. The infected alveolar macrophages by SARS-CoV-2 activate T cells (CD4^+^ and CD8^+^), which helps the release of interferon-γ. This facilitates tissue-resident alveolar macrophages to release cytokines, and further encourages the activation of T cells and form a positive loop. (Created with BioRender.com.)

Macrophages are another component of the innate immune system that works to resolve inflammation and repair the damaged tissues. There are two types of macrophages: alveolar macrophages (AMs) are proximate to alveolar type I and II epithelial cells (ATI and ATII); interstitial macrophages present in the parenchymal layer between the microvascular endothelium and the alveolar epithelium [118]. Alveolar macrophages are the body’s first line of protection against viral invasion and activation of the innate immune response. Both alveolar macrophages and interstitial macrophages contain two phenotypes: activated macrophages (M1 macrophages) and alternatively activated macrophages (M2 macrophages). The first one can recognize PAMP and be activated by Th1, while M2 is activated by T helper 2 cells (Th2). However, their functions are highly different. M1 possesses the capacity to initiate inflammatory response and secretes pro-inflammatory cytokines, hence it is called pro-inflammatory macrophages. On the other hand, the anti-inflammatory phenotype (M2) triggers anti-inflammatory cytokines and phagocytosis of apoptotic cells (efferocytosis) [119]. Recent study has revealed that M1 macrophages can promote viral replication and transmission, while M2 macrophages display opposite functions. This is probably because pH value of endosomes in M1 macrophages is lower than that in M2 macrophages, so it is helpful for virus to release RNA into cytoplasm and achieve replication. At the same time, lysosomes in M2 macrophages are more acidic compared with M1 macrophages, which is more favorable for viral degradation [120]. A great amount of pro-inflammatory chemokines and cytokines are released by macrophages, but with a limited type I interferon level [121]. BALF from COVID-19 patients includes high level of proinflammatory macrophages [95]. Lung macrophages from patients with severe infection show a higher level of cytokines and chemokines, such as IL-6, IL-1ß, TNF-α, CCL-2, CCL-3, and CCL-4 [95]. Similarly, another study indicates that monocyte-derived macrophages induce large amount of antiviral and proinflammatory cytokines, including IFN-α/β, TNF, IL-1β, IL-6, IL-10, and CXCL10 [122]. Although macrophages are an important component of innate immunity, they are also associated with adaptive immunity. A report found a positive feedback loop between SARS-CoV-2 containing macrophages and activated T cells that promote inflammation and subsequent injury [123]. The study noted that the virus first infects and replicates in the nasopharynx cells because of high levels of ACE2. SARS-CoV-2 then infects tissue-resident alveolar macrophages, which activates the recruitment of T cells to the alveolar region. The CD4^+^ and CD8^+^ T cells then produce interferon-γ; and subsequently, proinflammatory cytokines, including CXCL10, CCL4, CCL20, are released by tissue-resident alveolar macrophages to further activate T cells. They form a positive feedback loop (Fig. 6).

#### Natural killer (NK) cells

**Fig. 7:**
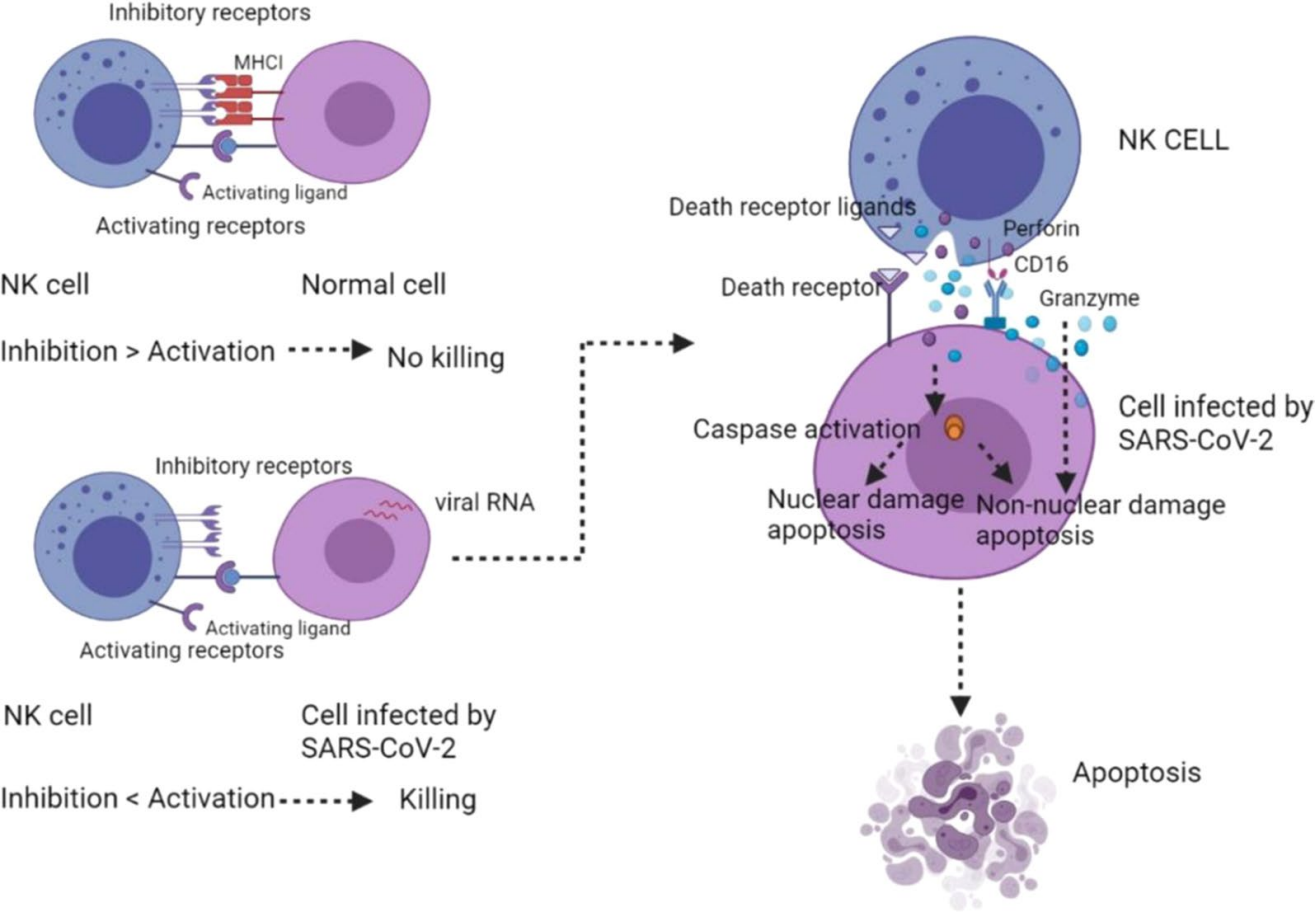
NK cells induce apoptosis in both direct and indirect ways [126, 127]. MHC expression on cells helps NK cells to determine killing or not. Lysis starts when the activating receptor is greater than inhibiting receptor. No killing is started in the opposite situation. In the first scenario, it further causes caspase dependent and independent pathways, which lead to nuclear damage or non-nuclear damage apoptosis. NK cells, natural killer cells; MHC, major histocompatibility complex. (Created with BioRender.com.)

Natural killer (NK) cells account for 5–20% of total circulating lymphocytes involved in the immune response against both virus invasion and cancer development. NK cells are one of the first cell types that reach the target inflammatory organs and respond rapidly [124]. They can directly kill the cells and produce cytokines for antiviral, such as INF-γ and TNF-α/β.[125] NK cells can eliminate pathogen-infected cells by apoptosis through both direct and indirect mechanisms [126]. For the direct pathway, the first problem encountered by NK cells is to distinguish between normal cells and virus-infected cells. This is achieved through activation and inhibition of the receptors. Normal cells contain MHC class I molecules, which act as a ligand for inhibitory response in NK cells. However, abnormal cells, including those infected by SARS-CoV-2, are probably be killed due to the lack of MHC class I [127]. Ligands expression of the activating receptors must be greater than that of inhibiting receptor for lysis to begin. Upon activation, NK cells release membrane-disrupting protein perforin and serine protease granzymes via exocytosis. Apoptosis is further induced with or without caspases. The caspase-independent apoptotic pathway is achieved through direct granzyme-mediated cell damage, which does not cause nuclear damage. However, the caspase-dependent apoptotic pathway can cause both nuclear damage and non-nuclear damage. In the case of non-nuclear damage, cell death still occurs without nuclear damage when caspases are inactivated [128, 129]. Moreover, apoptosis induced by the direct pathway is also achieved through death receptor-mediated cytotoxicity. Death occurs when the death receptor (Fas/CD95) on target cells recognizes the death ligands produced by NK cells with activated caspases. FasL and tumor necrosis factor-related apoptosis-inducing ligand (TRAIL) are two common types of death ligands [130]. On the other hand, antibody-dependent cellular cytotoxicity (ADCC) is an indirect recognition mechanism. When antibody binds to antigens on the target cells, CD16 on NK cells recognizes the antibody and induces apoptosis (Fig. 7) [131]. Significant reduction in NK cells has been found in severe patients infected with SARS-CoV-2 [132]. It has been found that NK cells display anti-SARS-CoV-2 effect, but are not functional in severe cases. The expression of IFN-α related genes are upregulated in NK cells in severe COVID-19 patients. However, the increased expression of TNF induced genes was discovered in moderate cases. Thus, the IFN-induced NK cells probably serve as a marker to indicate the adverse disease process [133]. Moreover, researchers found unconventional CD65^dim^ CD16^neg^ NK cells alongside with decreased NK cell cytotoxicity in PBMCs. In mild patients, the conventional CD65^dim^ CD16^pos^ NK cells appeared, and the cytotoxicity recovered in a short time. However, the process took longer in severe infected patients [134]. Patients with severe respiratory failure (SRF) may also show macrophage activation syndrome (MAS) with a prominent reduction in natural killer cells, CD19 lymphocytes, and CD4 lymphocytes [135]. A common feature of coronavirus infections is the substantial morbidity and mortality associated with exaggerated immune responses resulting in lung injury and acute respiratory distress syndrome, of which NK cells are an important component.

The main functions and effects of the above discussed cells are summarized in Table 1.

**Table 1:** The main cells involved in innate immunity and their biological functions in COVID-19 patients

Cell types	Major functions	Cell Responses in SARS-COV-2 infection	Severe cases VS mild	Cytokine release	References
Neutrophils	Kill pathogens in three main ways: phagocytosis, degranulation, and form neutrophil extracellular traps (NETs)	Increased level of NETs, which are related to ARDS	Contain immature neutrophils in severe patients Increased CD63^+^, CD274^+^ neutrophils in severe infected patients	IL-1β, IL-6, and CXCL8	Papayannopoulos [105] Middleton et al. [109] Schulte-Schrepping et al. [116] Neeland et al. [117] Vanderbeke et al. [113]
Macrophages	Resolve inflammation, repair damaged tissues, and trigger phagocytosis	High level of pro-inflammatory macrophages in BALF from COVID-19 patients	Higher level of cytokines and chemokines in lung macrophages from severe patients	IL-1ß, IL-6, IL-10, TNF-α, IFN-α/β, CCL-2, CCL-3, CCL-4 and CXCL10	Liao et al. [95] Zheng et al. [122]
NK cells	Use apoptosis to eliminate pathogen-infected cells and produce cytokines	Reduced NK cell levels	Significant reduction in NK cells in severe patients Upregulated NK cells expression of IFN-α related genes	Il-6, INF-γ and TNF-α/β	Bao et al. [126] Zheng et al. [132] Krämer et al. [133] Market et al. [125]

### Drugs that mediate inflammatory response

Searching for a cure for SARS-CoV-2 infected patients is urgent, but new drug development is time-consuming. Therefore, seeking commercialized drugs with potential curable effects is an ideal way to help patients and prevent disease spreading. Drugs that target innate immunity and inflammation are promising since the excessive inflammatory response is dangerous to patients. Moreover, it is found that anti-inflammatory drugs would cause less viral evolution that makes vaccines or other types of drugs more efficient [67]. In this review, we briefly introduce some drugs against inflammatory response caused by SARS-CoV-2 infection and discuss the merits and drawbacks as well.

#### Tocilizumab

**Fig. 8:**
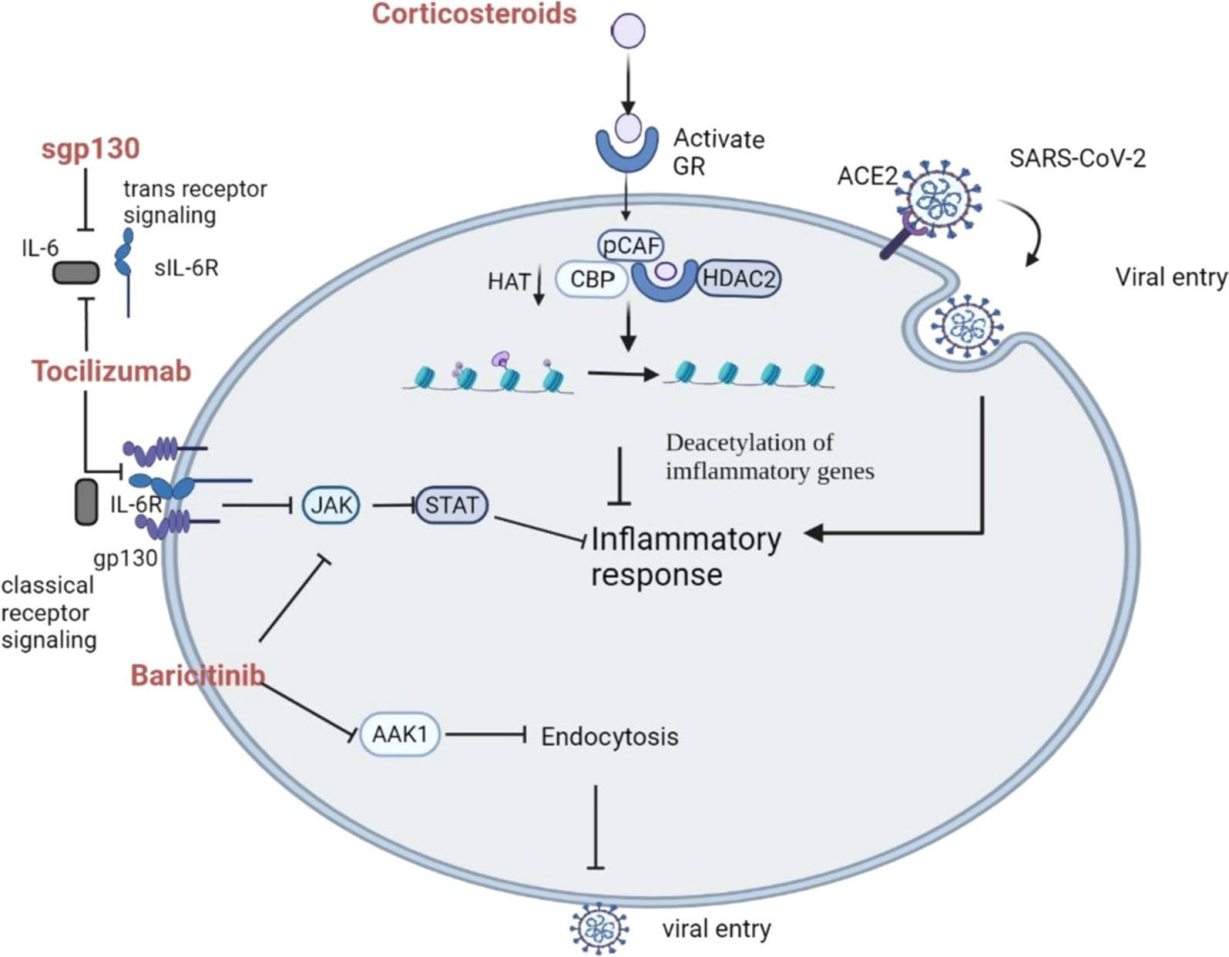
A diagram of anti-inflammation drugs against SARS-CoV-2 infection [138, 144]. When SARS-CoV-2 enters host cells and releases RNA, inflammatory response causes severe damage. Tocilizumab and sgp130 work together against IL-6, while Baricitinib targets JAK signaling pathway. The anti-inflammatory effect of corticosteroids is based on the activation of GR. sgp130, soluble gp130; GR, glucocorticoid receptors; HAT, histone acetyltransferase; HDAC2, histone deacetylase2; CBP, cAMP-response-element-binding-protein-binding protein, pCAF, p300/CBP-associated factor; JAK, Janus Kinase; STAT, signal transducer and activator of transcription; AAK1, AP2-associated protein kinase 1. (Created with BioRender.com.)

Tocilizumab is a humanized monoclonal antibody that works against IL-6 for the treatment of chronic inflammatory diseases. It can block IL-6/IL-6 receptor interactions and inhibits IL-6-mediated signal transduction subsequently (Fig. 8) [136]. Tocilizumab can influence IL-6 through two pathways: classical (membrane-bound) and soluble (trans) receptor signaling [137]. The first process is initiated by binding to IL-6R. Once the complex is formed, glycoprotein 130 (gp130) initiates the signaling via JAK and STAT, which lead to the activation of transcription factors. On the other hand, IL-6 can also bind to the sIL-6R. One of the different effects is that the trans-signaling pathway (mediated by sIL-6R) is presented as a pro-inflammatory signaling, while the other is an anti-inflammatory signal. In addition, the IL-6/sIL-6R complex can stimulate cells in other tissues without IL-6R, as gp130 is present in all types of cells in the body [138].

As shown above, IL-6 plays an important role in the cytokine storm after being affected by SARS-CoV-2. Therefore, it is worth considering and looking forward to whether tocilizumab could be a new approach for the treatment of COVID-19. Tocilizumab shows positive outcomes in reducing cytokine storm, vasopressor support, and mortality [139]. Early respiratory improvement was also observed in patients with high level IL-6, but those with severe COVID-19 but low IL-6 didn’t show better health conditions after treating tocilizumab. Other cytokine factors such as TNF-α and IL-1β are probably also involved in a highly inflammatory response. In such cases, medications that target these factors should be used in this situation [140]. Patients were found to have reduced lymphocyte levels after tocilizumab administration, while IL-6 levels were temporally increased for a few days [141].

However, modulating both IL-6 signaling pathways is difficult because it probably leads to unintended consequences. Therefore, soluble gp130 (sgp130) is considered because it only inhibits trans-signaling pathway. This is because IL-6 itself does not bind to sgp130, but the IL-6/sIL-6R complex binds to it [142]. The trans-signaling is more involved in the inflammatory response to disease and is also stronger than membrane-bound signaling [143]. Therefore, more experiments and trials on sgp130 should be conducted to understand its function in the treatment of COVID-19.

#### Corticosteroid

Corticosteroids are types of man-made steroid hormones commonly used to treat asthma and rheumatoid arthritis. It is now considered as a potential drug to mitigate the deleterious effects caused by COVID-19 because of its anti-inflammatory effect. This can be achieved because corticosteroids reverse the histone acetylation of activated inflammatory genes. Corticosteroids first bind to the glucocorticoid receptors (GR) and then bind to some coactivator molecules such as CBP and pCAF to regulate their histone acetyltransferase (HAT) activity. Moreover, histone deacetylase 2 (HDAC2) is recruited to activated GR. As a result, inflammatory genes are further repressed through histone deacylation [144]. In addition, there are several ways in which inhaled corticosteroids (ICS) may positively impact COVID-19 (Fig. 8). Firstly, gene expression of ACE2 and TMPRSS2 was found lower in asthmatics patients after receiving ICS [145, 146]. This finding indicates that corticosteroids can reduce viral replication, as both ACE2 and TMPRSS2 play critical roles in viral infection. In addition, corticosteroids have been shown to reduce the mortality in COVID-19 patients who developed ARDS, especially those who have elevated inflammatory markers such as methemoglobin [147, 148].

Dexamethasone is a type of corticosteroid that is used to treat COVID-19 in some cases. Treatment with dexamethasone increased survival time for patients with no mechanical ventilation in the first 28 days compared with standard care only [149]. Another study indicated that dexamethasone reduced 28-day mortality in patients requiring supplementary oxygen compared to usual care [150]. However, the risks of corticosteroids remain obscured. The Centers for Disease Control and Prevention (CDC) does not recommend the use of corticosteroids as a treatment for coronavirus because steroids may prolong viral replication in MERS patients. The immunosuppressive effect caused by long-term corticosteroid use also probably increases the risk of viral infection [151]. Moreover, corticosteroid therapy, including dexamethasone, can lead to euphoria in the short term and depression in the long term [152]. Higher mortality rate was even discovered in patients with COVID-19 after treating with corticosteroids compared to those who didn’t take them. This is probably because of the pro-thrombotic effect of steroids and other side effects [153]. On the other hand, it is important to control the doses of corticosteroids, as there is no specific evidence suggest high-dose treatments are effective.

#### Baricitinib

Baricitinib, a drug used for treating rheumatoid arthritis (RA), can reduce viral entry and inflammation. Baricitinib is an inhibitor of JAK1/JAK2. JAK binds to cytokine receptors and activates STATs, resulting in a pro-inflammatory response. JAK1 and JAK2 work together with type II cytokine receptors and contribute to IFNγ signaling [154]. Baricitinib influences IL-6 since JAK and STAT are also involved in the IL-6 signaling pathway (Fig. 8). In addition, baricitinib targets AP2-associated protein kinase 1 (AAK1) and cyclin G-associated kinase (GAK), which are regulators of endocytosis. Thus, baricitinib can prevent the virus from entering cells because the virus enters host cells via endocytosis (Fig. 8) [155, 156]. Rhesus Macaques treated with baricitinib showed several phenomena. Decreased level of inflammation and inflammatory cytokines has been displayed but type I IFN level is not impacted. Baricitinib also reduced the infiltration of macrophages, T cell levels, and NETosis [157]. For a small proportion of patients treated with baricitinib, their clinical characteristics and respiratory function parameters improve without serious adverse events [158]. However, baricitinib may have side effects that slow down a viral clearance and increase the risk of thromboembolic events due to its immunosuppressive property. In addition, administered to people who already have immunosuppressive properties or at inappropriate times, baricitinib probably impedes cytokines release and then delay immune restoration [159].

## Discussion and perspectives

The knowledge on SARS-CoV-2 infection and immune response are still limited. So far, studies on other viruses, including coronaviruses, could give rise to useful information for better understanding how the host responds to SARS-CoV-2 infection, and how the virus modulates innate immune response for the escape of host defense and induces pathogenesis. For example, Porcine delta-coronavirus Nsp5 inhibits IFN signaling by cleaving STAT2 [160]. Zika virus Nsp5 induces PIM1 expression that downregulates type I interferon level [161]. As an endoribonuclease (EndoU), Nsp15 of porcine epidemic diarrhea coronavirus (PEDV) is considered as a key factor for repressing type I IFN response, which helps viral replication and escape of host defense [162]. Nsp3 also serves as a potential research target for understanding how the virus antagonizes IFN-α/β signaling [72] Studies on Zika virus have revealed that a motif of NS3 can bind to RIG-I and MDA5 and prevent their translocation from cytosol to mitochondrial. The disruption of RIG-I and MDA5 signaling inhibits the downstream IFN-α/β induction [163]. Moreover, SARS-CoV papain-like protease (PLpro) is a domain of Nsp3 that represses IRF3 function by interacting with the STING-TRAF3-TBK1 complex [164]. It has also been revealed that SARS-CoV Nsp3 inhibits IRF3 phosphorylation [165]. Deubiquitinase (DUB) activity of PLpro is informed to be an important component for antagonizing interferon response [166, 167]. Although research has manifested that Nsp3 of SARS-CoV-2 has reduced DUB compared to SARS-CoV [74], another study showed an improved antiviral IFN-α/β response by treating with GRL-0617, an inhibitor of Nsp3 of SARS-CoV-2, [168] indicating an ideal target for antiviral drug development. We believe it is worthwhile for further in-deep studies. Since the above coronaviruses share similar traits with SARS-CoV-2, understanding their protein functions is not only helpful to comprehend SARS-CoV-2 activity; but also provides new insights for researchers to identify new targets for drug development. However, it is difficult to get a comprehensive picture of viral evasion strategies because several viral proteins probably work together to target the same host proteins, or one viral protein works on several pathways with different mechanisms. On the other hand, many factors can affect each other in SARS-CoV-2 studies, such as the strains of virus, time of viral infection, choice of cell lines, viral concentration, genotypes, etc. Thus, more research and carefully designed experiments are needed to elucidate the whole picture of viral evasion strategies.

## Conclusion

In this review, we have introduced the updated knowledge of SARS-CoV-2, including its structure, entry, and transmission processes. Then we have focused on inflammatory and innate immune responses caused by the virus, especially the cytokine storm. Type I interferon response is discussed, and the strategies of evading innate immune defense used by SARS-CoV-2 are emphasized. The viral proteins involved in antagonizing type I interferon response have also been discussed. We suggest that more studies should be conducted to better understand how SARS-CoV-2 employs multiple strategies to modulate host innate immunity and inflammatory response, because they play crucial roles in not only the virus survival and transmission, but also the disease progression and mortality of COVID-19 patients. This review would help us to figure out the current challenge questions of SARS-CoV-2 infection.

We have also reviewed the current promising drugs targeting innate immune and inflammatory responses. It is believed that cocktail therapy may probably give rise to a breakthrough. A combination of tocilizumab and corticosteroids has shown positive results for anti-inflammatory effects with a higher survival rate [169]. Early triple antiviral therapy with interferon beta-1b, lopinavir–ritonavir, and ribavirin has been conducted. The duration of hospital stays and the viral shedding became shorter after treating with such a combination therapy as compared to lopinavir–ritonavir treatment only [170]. Although the current outcomes of cocktail therapy above are promising, side effects still exist. More parameters should be considered in the clinic settings, such as the underlying health problems of patients, phase, timing, with or without mechanical ventilation, and doses of drugs etc. Studies should be continued to testify the safety and side effects of drugs to define the most appropriate COVID-19 therapy.

## Data Availability

Not applicable.

## Abbreviations

AAK1: AP2-associated protein kinase 1
ACE2: Angiotensin converting enzyme
ADAM17: Disintegrin and metalloprotease 17
ADCC: Antibody-dependent cellular cytotoxicity
ALRs: AIM2-like receptors
Amp: IL-6 amplifier
AMPK: AMP-activated protein kinase
Ang II: Angiotensin II
ARDS: Acute Respiratory Distress Syndrome
CARD: Caspase activation and recruitment domain
CBP: CAMP-response-element-binding-protein-binding protein
CLRs: C-type lectin receptors
ComC: Complement cascade
DAMPs: Damage-associated molecular patterns
DUB: Deubiquitinase
E protein: Envelope protein
EndoU: Endoribonuclease
ERGIC: ER-Golgi intermediate compartment
Gp130: Glycoprotein 130
GR: Glucocorticoid receptors
GRP78: Glucose regulated protein 78
GSDMD: Gasdermin D
HAT: Histone acetyltransferase
HDAC2: Histone deacetylase2
hnRNPs: Heterogeneous nuclear ribonucleoproteins
IKKɛ: Inhibitor of k-B kinase-ɛ
IRF: Interferon regulatory factor
M protein: Membrane protein
MAVS: Mitochondrial antiviral signaling protein
MDA5: Melanoma differentiation-associated protein 5
METTL3: Methyltransferase-like 3
MHC: Major histocompatibility complex
MPO: Myeloperoxidase
MYD88: Myeloid differentiation primary response protein 88
NE: Neutrophil elastase
NETs: Neutrophil extracellular traps
NF-κB: Nuclear factor-κB
NLRP3: NLR family pyrin domain containing 3
NLRs: NOD-like receptors
Nsp: Nonstructural protein
OAS-RNase L: Oligoadenylate synthetase–ribonuclease L
ORF: Open reading frames
PAD4: Peptidyl arginine deiminase 4
PAMPs: Pathogen-associated molecular patterns
pCAF: P300/CBP-associated factor
PKR: Protein kinase R
PRR: Pattern-recognition receptors
RAAS: Renin–angiotensin–aldosterone system
RBD: Receptor-binding domain
RdRp: RNA-dependent RNA polymerase
RLRs: RIG-I-like receptors
RNP: Ribonucleoprotein complex
ROS: Reactive oxygen species
S protein: Spike protein
sIL-6R: Soluble IL-6 receptor
STAT: Signal transducer and activator of transcription
TBK1: TANK binding kinase 1
Th17: Helper T17
TLR: Toll-like receptor
TMPRSS: Transmembrane serine protease
TNF: Tumor necrosis factor
TOM70: Translocases of outer membrane 70
